# Visual detection of porcine epidemic diarrhea virus by recombinase polymerase amplification combined with lateral flow dipstrip

**DOI:** 10.1186/s12917-022-03232-5

**Published:** 2022-04-18

**Authors:** Lei Ma, Kaiqi Lian, Mengjie Zhu, Yajie Tang, Mingliang Zhang

**Affiliations:** 1 School of Biotechnology and Food Engineering, Anyang Institute of Technology, Anyang, China; 2 Henan Joint International Research Laboratory of Veterinary Biologics Research and Application, Anyang, China

## Abstract

**Background:**

Porcine epidemic diarrhea virus (PEDV) is one of the most important enteric viruses causing diarrhea in pigs. The establishment of a rapid detection method applicable in field conditions will be conducive to early detection of pathogen and implementation of relevant treatment. A novel nucleic acid amplification method, recombinase polymerase amplification (RPA), has been widely used for infectious disease diagnosis.

**Results:**

In the present study, a reverse transcription (RT)-RPA assay combined with lateral flow dipstrip (LFD) was established for the visual detection of PEDV by targeting the N gene. The RT-RPA-LFD assay detected as low as 10^2^ copies/µL of PEDV genomic RNA standard. Moreover, the novel RT-RPA-LFD assay did not show cross-reactivity with common swine pathogens, demonstrating high specificity. The performance of the assay for detection of clinical samples was also evaluated. A total number of 86 clinical samples were tested by RT-RPA-LFD and RT-PCR. The detection results of RT-RPA-LFD were compared with those of RT-PCR, with a coincidence rate of 96.5%.

**Conclusion:**

The newly established RT-RPA-LFD assay in our study had high sensitivity and specificity, with a potential to use in resource-limited areas and countries.

## Background

Porcine epidemic diarrhea virus (PEDV) is an important enteric virus leading to tremendous economic losses to global swine industry [1–4]. PEDV infects pigs of all ages, causing severe diarrhea. The clinical signs of the infected pigs are vomiting, acute watery diarrhea, dehydration, and weight loss, with piglet mortality rates reaching 100% [5–7]. Porcine epidemic diarrhea (PED) caused by PEDV was first described in farm pigs in England in 1977 [8]. Since then, PEDV has spread across the world and has been isolated in many countries, including the USA, the UK, Argentina, Russia, and China, resulting in heavy economic losses to porcine industry [3, 7, 9–11].

Up to now, besides PEDV, three other swine coronaviruses associated with intestinal diseases have been identified: transmissible gastroenteritis virus (TGEV), porcine deltacoronavirus (PDCoV), and swine acute diarrhea syndrome coronavirus (SADS-CoV) [12–14]. The clinical symptoms of the intestinal diseases caused by these swine coronaviruses are highly similar. Thus, it is difficult to distinguish PEDV infection from other swine coronaviruses by the clinical signs of the infected pigs. Therefore, a rapid and robust diagnostic method for the detection of PEDV is of vital importance for the identification of the source of infection, which would be helpful for the implementation of specific control programs.

In recent years, many research efforts have been focused on the development of the point-of-care (POC) tools that can be utilized in field conditions for the diagnosis of infectious diseases [15]. The POC methods or devices would allow etiological diagnosis in the rural farms or resource-limited settings. Many POC tests have been developed for the detection of swine infectious diseases based on isothermal nucleic acid amplification technologies, including loop-mediated isothermal amplification (LAMP), recombinase polymerase amplification (RPA), and nucleic acid sequence-based amplification (NASBA) [16–18]. RPA is an effective technique that has been successfully employed to detect various pathogens in clinical samples with outstanding performance [19]. In a RPA assay, recombinase enzymes scan for homologous sequences of specific primers in a DNA template. Amplification is then extended by a polymerase from the primer-binding site at a constant temperature. Generally, up to 30 min are needed for completion of exponential amplification. The main endpoint detection methods for RPA products are agarose gel electrophoresis, fluorescent probe, and lateral flow dipstrip (LFD) [20–22]. RPA assay combined with LFD requires minimum laboratory equipment and has the potential to implement in resource-limited areas. The colored band on the LFD gives a straightforward result interpretation.

To date, no reverse transcription-RPA-LFD (RT-RPA-LFD) assay for visual detection of PEDV was reported. The aim of the present study was to establish a RT-RPA-LFD assay specific for PEDV detection.

## Results

### Optimized RT-RPA-LFD conditions

**Fig. 1:**
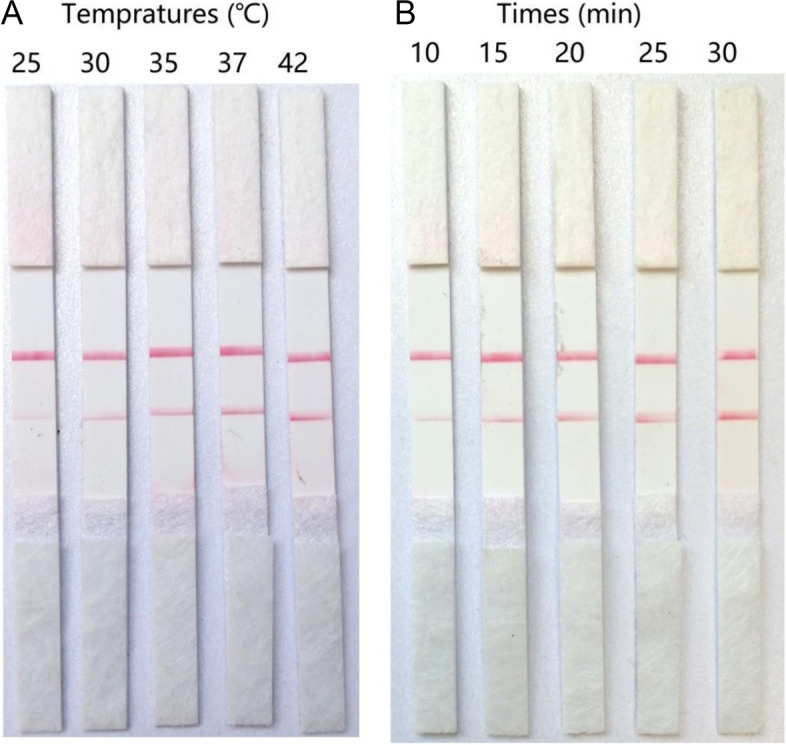
Optimization of the RT-RPA-LFD assay (**A**) Different incubation temperatures (25℃, 30℃, 35℃, 37℃, and 42℃) and (**B**) times (10 min,15 min,20 min,25 min,and 30 min) were assessed

RT-RPA tests were performed at different incubation temperatures (25 °C, 30 °C, 35 °C, 37 °C, 42 °C) and incubation times (10, 15, 20, 25 and 30 min). The optimized reaction conditions were determined by the color density of the test line. At an incubation time of 30 min, the color densities of the test lines of the RPA products at 37 °C and 40 °C were the same. Therefore, a temperature of 37 °C, which is closer to that of the human body, was chosen as the optimal reaction temperature. When the incubation temperature was set as 37 °C, the color of RPA products that incubated for 30 min was the most obvious on LFD. Taken together, an incubation temperature of 37 °C and an incubation time of 30 min were determined as the optimal reaction conditions for performing the novel RT-RPA-LFD assay (Fig. 1).

### Detection limit of RT-RPA-LFD

**Fig. 2:**
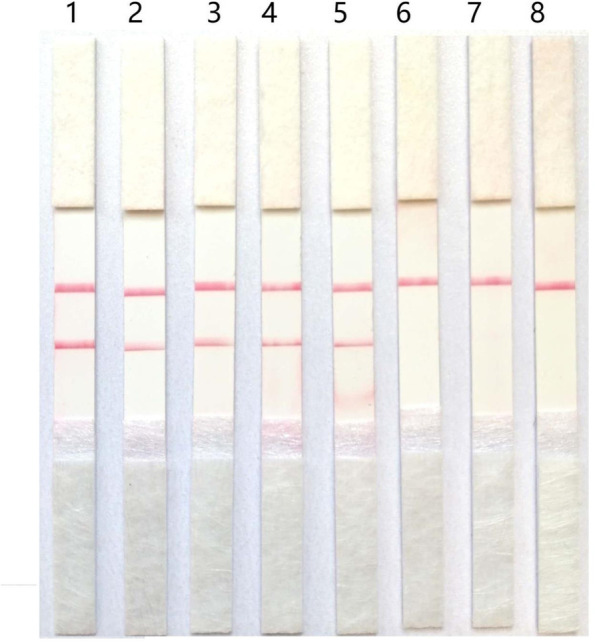
Sensitivity of the RT-RPA-LFD assay. To determine the detection limit of the RT-RPA-LFDassay, tenfold serial dilutions of the RNA standard ranging from 10^6^copies/µL to 1 copy/µL were tested. 1–7:10^6^, 10^5^, 10^4^, 10^3^, 10^2^, 10, and 1; 8: Distilled water

Seven RNA dilutions (10^6^, 10^5^, 10^4^,10^3^,10^2^,10^1^, and 10^0^ copies/μL) were detected by the RT-RPA-LFD assay in triplicate. As can be seen in the Fig. 2, these RNA dilutions (10^6^,10^5^,10^4^,10^3^,10^2^ copies/μL) were PEDV-positive. However, the RNA dilutions of 10 copies/μL and 1 copy/μL were PEDV-negative. Distilled water also yielded a negative result. All tests were performed in triplicate and the same results were obtained.

### Specificity of RT-RPA-LFD

**Fig. 3:**
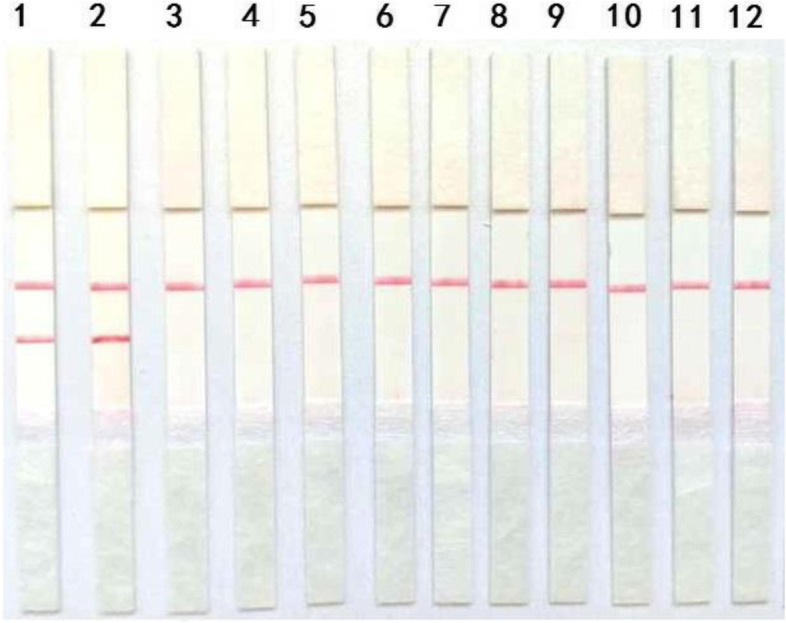
Specificity test of the RT-RPA-LFD assay. The nucleic acids of TGEV, RV, PRRSV, CSFV, PPV, FMDV, PCV2, PCV3 and PRV were used to validate the cross-reactivity of the RT-RPA-LFD assay. 1–2: PEDV wild strain and vaccine strain, 3–10: TGEV, RV, PRRSV, CSFV, PPV, FMDV, PCV2, PCV3, PRV, distilled water

Pathogens that commonly infect pigs were subjected to the RT-RPA-LFD assay. As is visible in Fig. 3, PEDV wild strain and vaccine strain were positive. The other pathogens (TGEV, RV, PRRSV, CSFV, PPV, FMDV, PCV2, PCV3 and PRV) and distilled water produced negative results.

### Validation of clinical detection

Eighty-six clinical samples collected from the piglets that were suspected to have been infected with PEDV were tested by the RT-RPA-LFD assay. Thirty-nine of them were PEDV-positive, whereas the other ones were negative. RT-PCR tests of all samples showed that42of them were PEDV-positive, and 44 samples were PEDV-negative. All PEDV-positive samples analyzed by RT-PCR were also found to be positive by RT-RPA-LFD. However, 3 samples that were PEDV-positive by RT-RPA-LFD testing were found to be negative by RT-PCR. The Se (sensitivity) and Sp (specificity) of the RT-RPA-LFD assay were 92.9% and 100% separately (Table 1). The coincidence rate of the RT-RPA and RT-PCR test results was 96.5%, indicating the RT-RPA-LFD assay was effective for detection of PEDV (Table 1).

**Table 1:** Comparison of RT-RPA-LFD and RT-PCR

	RT-PCR			CR	Se	Sp
	Positive	Negative	Total			
RT-RPA-LFD						
Positive	39	0	39	96.5%	92.9%	100%
Negative	3	44	47			
Total	42	44	86			

CR: Coincidence rate. CR = (39 + 44)/86*100%. Se = 39/42 = *100%. Sp = 44/44*100%

## Discussion

PEDV is considered one of the most important viruses causing gastrointestinal symptoms in pigs, contributing to heavy economic losses in swine industry worldwide. To date, several diagnostic methods for the detection of PEDV have been developed based on nucleic acid detection technologies, such as RT-PCR, cross-priming amplification (CPA), and reverse-transcription loop-mediated isothermal amplification (RT-LAMP) [23–25]. Due to its high specificity and sensitivity, RT-PCR has become the most commonly used nucleic acid detection method [24]. Furthermore, multiplex real-time RT-PCR assay was also developed to discriminate classical PEDV from variant strains using two primer–probe sets targeting the S gene [24]. However, the sophisticated laboratory equipment and well trained staff are required to perform RT-PCR assays. Besides, two primer–probe sets made result judgment difficult, the cost of the test also rise.

RPA technology was invented in 2006 but has experienced rapid development in the last years [19, 26]. The RPA technology had several advantages over PCR: constant reaction temperature; less equipment requirement (a metal or water bath); shorter reaction time (less than 30 min). Therefore, many diagnostic methods based on RPA technology have been developed to detect various human and animal pathogens. Generally, a RPA product is detected by a fluorescence detector or a LFD [18, 27–30]. Real-time detection of PEDV by the RT-RPA assay has been reported [31]. Nevertheless, a fluorescence detector is still required for conducting the real-time RT-RPA assay. In our study, only two labeled primers were used. The RT-RPA reaction generated a dual-tagged DNA amplicon which could be visualized on a commercial LFD which is labeled with the anti-FITC antibody [32, 33]. To avoid false positive result in the RT-RPA-LFD assay, some solutions were adopted: open and close the reaction tubes carefully, change the gloves frequently, and perform the RT-RPA assay and LFD detection in different places.

The RT-RPA-LFD assay established in present study demonstrated remarkable sensitivity and specificity. The assay could detect 100 PEDV genomic copies and showed no cross-reactivity with other common porcine pathogens. The assay was performed at 37 °C in a metal bath. The target of the primers for the RT-RPA-LFD assay was the conserved N gene sequence. The sequence of the primers shared 98%–100% similarity with the archived data. We found that the assay detected almost all circulating PEDV strains, including the attenuated vaccine strain. In the 42 PEDV-positive samples confirmed by a RT-PCR assay in our study, 38 samples were positive for the wild PEDV strains, whereas 4 samples were positive for the attenuated vaccine strain by the RT-PCR assay. A previous study reported that a total number of 80 suspected PEDV samples were assessed by real-time RT-PCR assay and conventional RT-PCR assay, yielding positive rates of 81.25% and 77.50% for PEDV wild-type strains, and 8.75% and 7.50% for PEDV attenuated vaccine strains, respectively [34].

Although the RT-RPA-LFD assay could not discriminate the wild strains from the attenuated vaccine strain, the test results in our present study and previous study both showed that the wild-type strains make up the large majority of the clinical samples, whereas the number of the clinical samples containing the attenuated vaccine strains was very limited. Rapid detection of the infectious agent upon the appearance of clinical symptoms of swine diarrhea in a pig farm is of vital importance for the early implementation of treatment measures. That is especially important for farms in rural resource-limited areas. Discrimination the wild strains from an attenuated vaccine strain is less important than the rapid diagnosis of the infectious agent. The newly established RT-RPA-LFD assay needed only a metal bath and a portable LFD. However, the real-time RT-RPA assay requires a portable fluorescence detector, which costs approximately 3000 US dollars. Thus, the RT-RPA-LFD assay is more cost-effective and easier to perform compared with the real-time RT-RPA assay.

In summary, a RT-RPA-LFD assay targeting the highly conserved N gene was established for the rapid detection of PEDV. The RT-RPA method combined with LFD is an effective POC diagnostic tool, in which only a metal bath and LFD were sufficient for nucleic acid amplification and detection. The developed RT-RPA-LFD assay is extremely rapid and convenient to operate in field conditions, which offers considerable potential for PEDV monitoring and control in under-equipped settings.

## Materials and methods

### Preparation of RNA standard

A PEDV RNA standard was prepared for the establishment of the RT-RPA-LFD assay. PEDV RNA genome was extracted from the clinical intestine specimen that was PEDV-positive and was confirmed by conventional RT-PCR [35]. PEDV cDNA was prepared by PrimeScript™ 1st Strand cDNA Synthesis Kit (Takara, Beijing, China) according to the instructions. A primer pair specified for the PEDV N gene sequence, with a forward primer: 5’-AGGACTCGTACTGAGGGTGTT-3’ and a reverse primer:5’-ATGTTACACCACCACGGTCAT-3’, was designed based on the nucleotide sequence of a PEDV strain CV777 and synthesized by Sangon Biotech (Shanghai, China). Then, the N gene sequence was obtained using the cDNA as a template in a conventional PCR assay conducted by Premix Taq™ (Ex Taq™ Version 2.0 plus dye) (Takara, Beijing, China) following the manufacturer's guidelines. The specific PCR reaction conditions were as follows: 94 °C for 5 min; 94 °C for 30 s, 55 °C for 30 s, 72 °C for 30 s, 35 cycles; and 72 °C for 5 min. The obtained PCR products were subjected to electrophoresis on 2% agarose gel in Tris–acetate-EDTA (TAE) buffer and purified by TaKaRa MiniBEST Agarose Gel DNA Extraction Kit (Takara, Beijing, China) following the instructions. The purified amplicons were inserted into the pGEM-T vector (Promega Corporation, Madison, WI, USA) following the instructions and transformed into TOP10 competent cells. The positive recombinant plasmid was next confirmed by sequencing and linearized by endonuclease Pst I. Further, the linearized DNA was in vitro*-*transcribed by MAXIscript™ (MAXIscript; Ambion, Austin, TX, USA) following the manufacturer’s instruction. The in vitro*-*transcribed RNA was digested by DNase I to remove the residual DNA, purified by trizol(Thermo Fisher Scientific, Waltham, MA, USA) and preserved in TE buffer (Solarbio, Beijing, China). RNA standard was quantified using a NanoDrop 2000c spectrophotometer (Thermo Scientific, USA). The concentration of the RNA standard (copies/μl) was calculated using the following equation: RNA copy number (copies/μl) = (6.02 × 10^23^) × (RNA concentration (ng/μl) × 10^–9^)/(RNA length × 340).

### RT-RPA-LFD assay

**Fig. 4:**
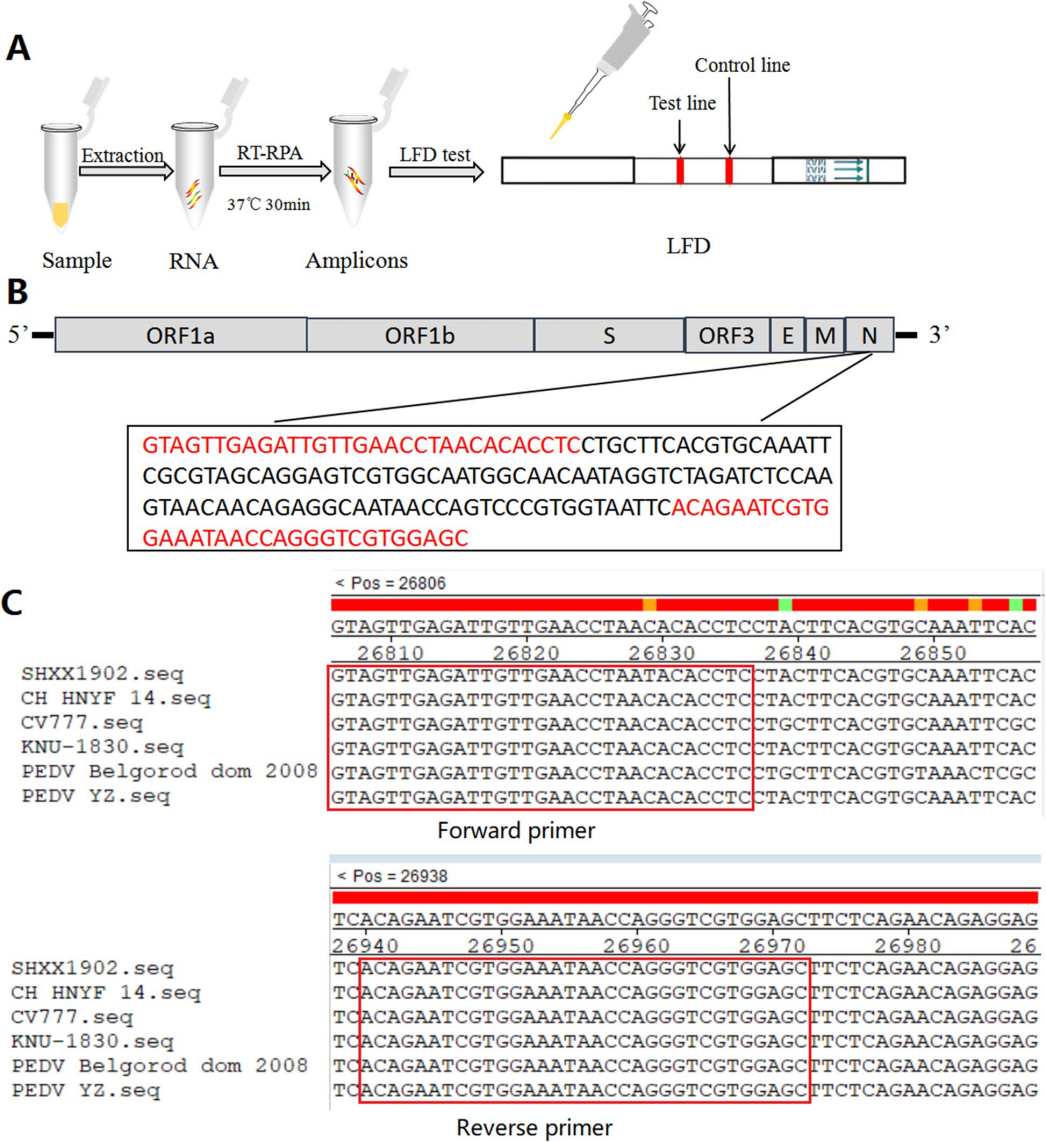
Schematic representation of the RT-RPA-LFD assay for the detection of PEDV. **A** Workflow of the RT-RPA-LFD assay. RNA extraction from samples can be used as the template the RT-RPA assay. The amplicons can be visualized by a LFD by naked eye. **B** Genome map showing the target sequence and primers. **C** Alignment of the target sequences of several PEDV strains. The positions of RT-RPA-LFD primers are indicated in the red box

The RT-RPA reaction was performed by TwistAmp Basic RT RPA Kit (TwistDx, Cambridge, UK) following the instructions in the manual. The enzymes used for the RT-RPA reaction were provided in the form of freeze-dried powder in tubes. The following ingredients were transferred into the tube to a final volume of 50μL: 29.5μL of rehydration buffer, 2.1 μL of each primer (10 μm), 1 μL of the template, and 12.8μL of water. Finally, 2.5μL of magnesium acetate (280 mM) was added onto the tube lids. Then, the tube lids were immediately closed and centrifuged briefly. The tubes were next placed in a metal bath and incubated at 37 °C for 30 min. Then, the RT-RPA products were 1:100 diluted with buffer and added on LFD (BioUSTAR, Hangzhou, China). A visual band on the control line of LFD confirmed the validity of the result. A visual band on the test line showed that the sample was PEDV-positive, and no visual band on the test line indicated that a negative result. The lack of a visual band on the control line showed an invalid result, and the test had to be repeated. The workflow of the RT-RPA-LFD assay was shown in Fig. 4A.

### RT-RPA-LFD primer design

To date, no computer software is available for automatically designing RPA primers. The guidelines provided by TwistDx Inc. (Cambridge, UK;www.twistdx.co.uk) for primer design were employed in our study. The main points were as follows: The primer length was within 30–35 nucleotides (nt); oligonucleotides that contained sequence elements promoting secondary structures and primer-primer interactions or hairpins had to be discarded. The amplification product length was within 100–200 bp. More detailed information concerning the primer and probe design could be found in the Assay Design Manual provided by TwistDx. The following primer pair was designed in compliance with the instructions and synthesized by Sangon Biotech (Shanghai, China): forward primer: FITC-GTAGTTGAGATTGTTGAACCTAACACACCTC, reverse primer: Biotin-GCTCCACGACCCTGGTTATTTCCACGATTCTGT. The sequence of the RT-RPA amplicons was shown in Fig. 4B. Alignment of the primers of several PEDV strains was performed (Fig. 4C).

### Optimization of the reaction conditions

The RT-RPA reaction could be successfully performed within a broad range of reaction temperatures and incubation times. Evaluation of the amplification efficiency under different conditions would improve the performance of the RT-RPA-LFD assay. Thus, the RT-RPA tests in this examination were carried out at different incubation temperatures (25 °C, 30 °C, 35 °C, 37 °C, 42 °C) and incubation times (10, 15, 20, 25, and 30 min). The RT-RPA products were separately detected by LFD using the above-described procedure.

### Sensitivity and specificity evaluation

The RNA molecule standard was diluted with TE buffer and adjusted to 10^7^ copies/μL. The RNA dilution (10^7^ copies/μL) was ten-fold serially diluted with TE buffer. Seven RNA dilutions (10^6^, 10^5^, 10^4^,10^3^,10^2^,10^1^, and 10^0^ copies/μL) were detected by the RT-RPA-LFD assay in triplicates to determine the detection limit of the assay. Pseudorabies virus (PRV) was isolated and preserved in our laboratory as previously described [36]. TGEV H, PEDV CV777, and porcine rotavirus (RV) NX were purchased from Harbin Weike Biotechnology Ltd.(Harbin, China). Porcine reproductive and respiratory syndrome virus (PRRSV) strain JXA1 was purchased from China Animal Disease Control Center, classical swine fever virus (CSFV) strain C attenuate vaccine, porcine parvovirus (PPV) CP-99 killed vaccine, and pig foot-and-mouth disease virus (FMDV) killed vaccine were purchased from commercial vaccine companies. The nucleic acids of porcine circovirus type 2 (PCV2), porcine circovirus type 3 (PCV3) and pseudorabies virus (PRV) were detected in clinical samples, isolated, and preserved in our laboratory. The nucleic acids of these swine pathogens were next detected by the RT-RPA-LFD assay. PEDV wild strain was isolated and preserved in our lab. TE buffer served as a negative control, whereas PEDV genomic RNA isolated from PEDV wild strain was used as a positive control.

### Determination of the performance of the assay on clinical samples

The applicability of the RT-RPA-LFD assay for detection of PEDV was validated by 86 clinical samples collected from piglets with clinical signs of watery diarrhea and dehydration in Henan Province between April 2018 and February 2020. The homogenates of the samples were prepared as a 10% (w/v) suspension using phosphate buffered saline (PBS, pH 7.4). The intestinal samples were homogenized using a disposal pestle and centrifuged for 5 min at 8000r/s. The supernatant was then collected for RNA extraction by RNAeasy™ Animal RNA Extraction Kit (Beyotime, Shanghai, China) according to the instruction manual. Two μL of the nucleic acids was used as the template and tested by the above RT-RPA-LFD procedure. The clinical samples were also assessed by a conventional RT-PCR assay. The primers that could discriminate the field strains and attenuated strains had been previously reported [35]. The conventional RT-PCR assay was performed in a 50μL volume by One Step PrimeScript™ RT-PCR Kit (Takara, China).The test result of the RT-RPA-LFD was compared with that of RT-PCR.

## Data Availability

The data are available upon reasonable request from the corresponding author.

## Abbreviations

PEDV: Porcine epidemic diarrhea virus
RPA: Recombinase polymerase amplification
TGEV: Transmissible gastroenteritis virus
PDCoV: Porcine deltacoronavirus
SADS-CoV: Swine acute diarrhea syndrome coronavirus
CSFV: Classical swine fever virus
PPV: Porcine parvovirus
FMDV: Foot-and-mouth disease virus
PCV2: Porcine circovirus type 2
PCV3: Porcine circovirus type 3
PRV: Pseudorabies virus
